# Evaluation of the effect of trendelenburg position duration on intracranial pressure in laparoscopic hysterectomies using ultrasonographic optic nerve sheath diameter measurements

**DOI:** 10.1186/s12871-024-02624-4

**Published:** 2024-07-15

**Authors:** Hulya Guloglu, Dilek Cetinkaya, Tufan Oge, Ayten Bilir

**Affiliations:** 1 Department of Anesthesiology and Reanimation, Eskisehir Osmangazi University Medical Faculty, Eskisehir, Türkiye; 2 Department of Obstetrics and Gynecology, Eskisehir Osmangazi University Medical Faculty, Eskisehir, Türkiye

**Keywords:** Cerebral oxygenation, Pneumoperitoneum, Trendelenburg position

## Abstract

**Background:**

During laparoscopic surgery, pneumoperitoneum and Trendelenburg positioning applied to provide better surgical vision can cause many physiological changes as well as an increase in intracranial pressure. However, it has been reported that cerebral autoregulation prevents cerebral edema by regulating this pressure increase. This study aimed to investigate whether the duration of the Trendelenburg position had an effect on the increase in intracranial pressure using ultrasonographic optic nerve sheath diameter (ONSD) measurements.

**Methods:**

The near infrared spectrometry monitoring of patients undergoing laparoscopic hysterectomy was performed while awake (T0); at the fifth minute after intubation (T1); at the 30th minute (T2), 60th minute (T3), 75th minute (T4), and 90th minute (T5) after placement in the Trendelenburg position; and at the fifth minute after placement in the neutral position (T6). Results: The study included 25 patients. The measured ONSD values were as follows: T0 right/left, 4.18±0.32/4.18±0.33; T1, 4.75±0.26/4.75±0.25; T2, 5.08±0.19/5.08±0.19; T3, 5.26±0.15/5.26±0.15; T4, 5.36±0.11/5.37±0.12; T5, 5.45±0.09/5.48±0.11; and T6, 4.9±0.24/4.89±0.22 ( *p* < 0.05 compared with T0). ). No statistical difference was detected in all measurements in terms of MAP, HR and ETCO2 values compared to the T0 value (*p* > 0.05).

**Conclusions:**

It was determined that as the Trendelenburg position duration increased, the ONSD values ​​increased. This suggests that as the duration of Trendelenburg positioning and pneumoperitoneum increases, the sustainability of the mechanisms that balance the increase in intracranial pressure becomes insufficient.

**Trial registration:**

This study was registered at Clinical Trials.gov on 21/09/2023 (registration number NCT06048900).

## Introduction

With advancements in technology, laparoscopic procedures have become more preferred than open surgical interventions. The primary considerations in selecting the laparoscopic approach are reduced risk of bleeding, less invasiveness, faster postoperative wound healing, and speedier discharge [1]. To provide a better surgical view during laparoscopic surgery, gas is introduced into the peritoneum and pneumoperitoneum, and the patient is placed in the Trendelenburg position. However, these two applications cause cardiovascular and respiratory physiological changes in the patient and elevate the intracranial pressure (ICP) [2]. The hydrostatic pressure difference caused by the head-down position causes an increase in both central venous pressure and arterial pressure. Pneumoperitoneum applied at the same time also contributes to these increases. Increased intra-abdominal pressure prevents venous return from the lumbar venous plexus. All of these increase the hydrostatic pressures within the cerebral vascular system, resulting in increased intracranial pressure. [3, 4].

While invasive measurements remain the gold standard for measuring ICP, due to the challenges and complications involved, attempts have been made to seek non-invasive methods [5]. One of these non-invasive methods is the ultrasonographic evaluation of optic nerve sheath diameter (ONSD). Many studies have shown that OSND is effective in promptly detecting increased intracranial pressure (ICP) by bedside evaluation [5–7].

The healthy human brain is able to regulate cerebral blood flow under a wide cerebral perfusion pressure. This regulation, defined as cerebral autoregulation, regulates cerebral blood flow through vasoconstriction or vasodilation. However, adaptation to the redistribution of blood in the vessels and cerebrospinal fluid (CSF) in the subarachnoid spaces and ventricles during postural changes is possible with the activation of many different adaptive mechanism [8]. Therefore, it has been reported that ICP is kept constant during pneumoperitoneum in the Trendelenburg position in laparoscopic surgery [9]. However, as the duration of Trendelenburg and pneumoperitoneum increases, questions remain regarding the sustainability of these adaptive mechanisms [8]. Thus, in this study, we aimed to investigate whether the Trendelenburg position duration had an effect on the increase in ICP using the ultrasonographic measurement of ONSD.

## Methods

### Patients

After receiving approval from the local ethics committee (02.03.202/15), this single-center, prospective, observational clinical study was registered at ClinicalTrials.gov (NCT06048900). Informed verbal and written consent was obtained from the patients. The study included 25 female patients aged over 18 with an American Society of Anesthesiologists (ASA) physical classification score of I-III who underwent total laparoscopic hysterectomy. Patients with known chronic lung disease, pulmonary hypertension, glaucoma, a history of ocular or intracranial surgery, diabetic retinopathy, intracranial masses, hydrocephalus, or an ASA score of IV, optic neuritis, and those who did not provide consent for participation in the study were excluded.

### Anesthesia protocol

In the preoperative evaluation, the patients’ demographic data (age, ASA score, body mass index [BMI]) and operation data were recorded. After the patients were transferred to the operating table, routine standard monitoring (electrocardiogram, pulse oximetry, and non-invasive blood pressure measurement) and near infrared spectrometry (NIRS) monitoring (INVOS 5100 C Cerebral/Somatic Oximeter, Medtronic, USA) were undertaken. The standard monitoring and NIRS parameters (rSO2) of the patients were recorded at 10-minute intervals. Following preoxygenation, general anesthesia was induced in the patients with intravenous (iv) propofol (2–3 mg/kg), remifentanil (1 mcg/kg), and rocuronium (0.5 mg/kg). After endotracheal intubation, anesthesia maintenance was provided with a 50% oxygen/air mixture with remifentanil at 0.5-1 mcg/kg/minute and sevoflurane at a 1-1.1 minimal alveolar concentration. Fluid therapy and sevoflurane concentration were adjusted to ensure that the mean arterial pressure (MAP) was not above 80 mmHg and rSO2 was not below 20% of the value obtained while awake. The aim was to maintain end tidal carbon dioxide (EtCO2) within the range of 35–40 mmHg by adjusting ventilation to 6–8 ml/kg and positive end-expiratory pressure (PEEP) to 5–8 cm/H2O. After trocars were placed into the abdomen and pneumoperitoneum (insufflation pressure: 12 mmHg) was applied, the patient was placed in the Trendelenburg position, with the side of the table where the patient’s head was situated inclined downward at an angle of 40–45 degrees. At the end of the surgery, pneumoperitoneum and Trendelenburg position were terminated. For postoperative analgesia, 1 g of paracetamol and 1 mg/kg of tramadol hydrochloride were administered intravenously to the patients. After the surgical procedure was completed, 2 mg/kg of suggammadex was administered to reverse neuromuscular blockade. Once it was confirmed that protective airway reflexes had been restored, the patients were extubated. The patients were monitored for eight hours in the recovery room and then transferred to the ward.

### Ocular sonography

Prior to the induction of anesthesia, the patients were in a neutral position with a clear antimicrobial drape placed over their eyes. ONSD measurements were performed in the sagittal and transverse planes in both eyes using a linear ultrasound probe (GE Healthcare Vivid-i, USA) without applying high pressure. All measurements were made by the same anesthesiologist. ONSD measurement was made from the section where the best image was taken after the optic disc was imaged. The best image was obtained by imaging at least 3 times for each plane. ONSD was measured 3 mm behind the optic disc. The values measured in the sagittal and transverse planes were averaged and noted separately for each eye (right eye T0R, left eye T0L).

The second measurement was taken at the fifth minute following endotracheal intubation in the neutral position (T1R-T1L). Following placement in the Trendelenburg position, bilateral ONSD measurements were taken at the 30th minute (T2R-T2L), 60th minute (T3R-T3L), 75th minute (T4R-T4L), and 90th minute (T5R-T5L). The last measurement was taken five minutes after the patient was placed in the neutral position (T6R-T6L).

### Statistical analysis

All statistical analyses were performed using the IBM SPSS v. 25.0 statistical program. In the statistical analysis of the data, continuous data were given as mean ± standard deviation and categorical data as percentages. Shapiro Wilk’s test is used to investigate the suitability of the data for normal distribution; ANOVA (One Factor Repetition) test was used for repeated measurements, and “Two way reapplied measurements ANOVA (One Factor Repetition)” test was used for two-way repeated measurements. The relationship between quantitative variables obtained from the patients’ clinical findings was examined with the Pearson correlation analysis. When calculating the sample size, the error rate was taken as 5% (α: 0.05) the power rate as 80%, and the sufficient sample size was found to be 19 patients. Statistical significance was evaluated at a p level of < 0.05.

## Results

Twenty-five patients who met the criteria were included in the study. The mean age of the patients was found to be 48.8 ± 10.16 years, and their mean BMI was 28.69 ± 4.38. Seven patients were evaluated as having an ASA score of I, while the remaining 18 had an ASA score of II (Table 1).

**Table 1:** Demographic variables

*N*:25	Mean ± SD
Age (yr) BMI ASA Class (I/II)	48.8 ± 10.16 28.69 ± 4.38 1.72 ± 0.46

BMI: Body mass index, ASA: American Society of Anesthesiologists

**Fig. 1:**
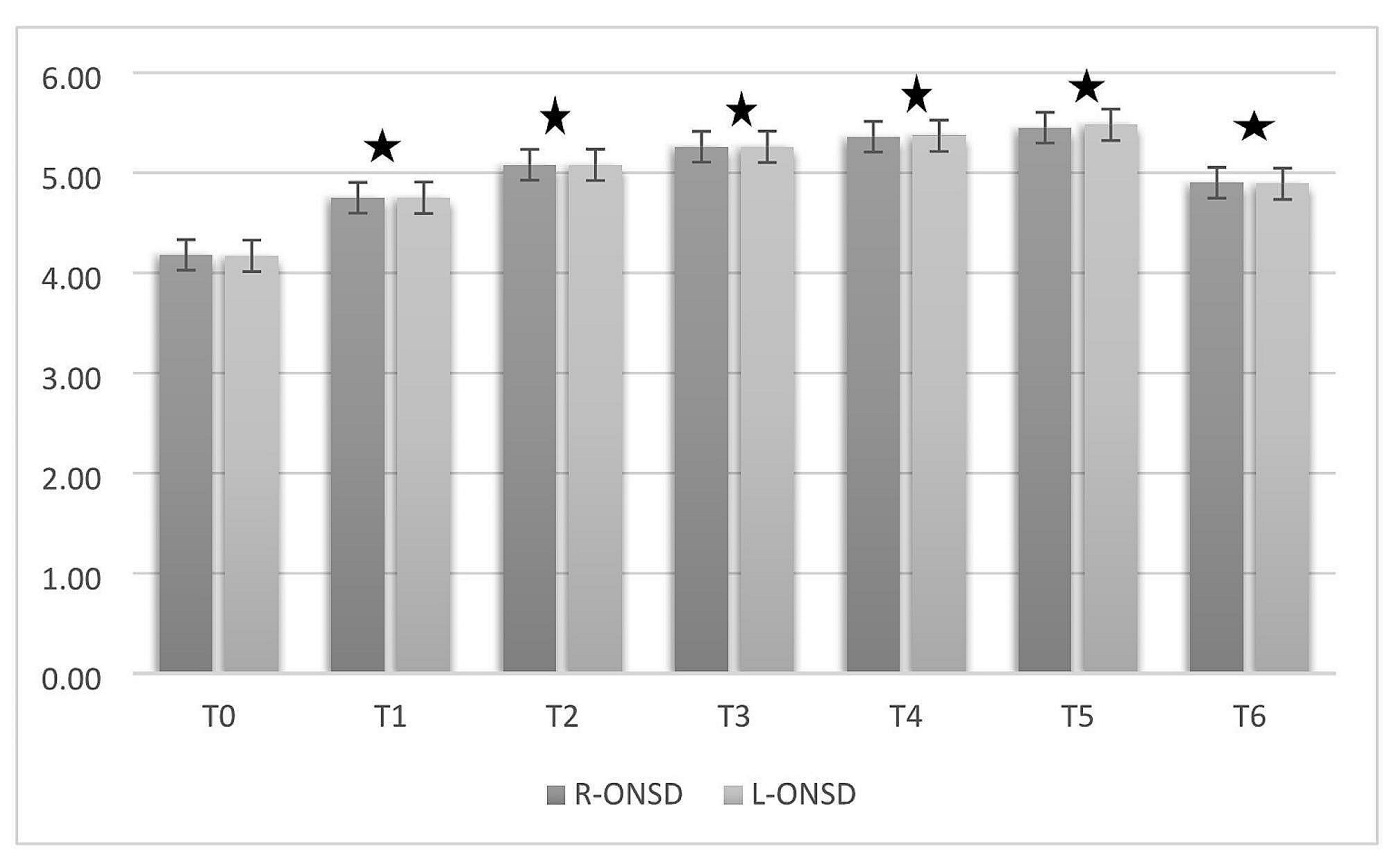
Mean values of optic nerve sheath diameter (ONSD) for the right and left eyes R-ONSD: Right eye-optic nerve sheath diameter, Left-ONSD: Left eye-optic nerve sheath diameter, T0. baseline (before anesthesia induction), T1. the fifth minute following endotracheal intubation in the neutral position, T2. 30 min after introducing pneumoperitoneum and Trendelenburg position, T3. 60 min after introducing pneumoperitoneum and Trendelenburg position, T4. 75 min after introducing pneumoperitoneum and Trendelenburg position, T5. 90 min after introducing pneumoperitoneum and Trendelenburg position, T6. five minutes after the patient was placed in the neutral position; * *P* < 0.05 compared with T0

Figure 1 shows the ONSD measurements There was no statistically significant difference in the comparisons made for each value between the two eyes (*p* > 0.05). The measured ONSD values were as follows: T0 right/left, 4.18±0.32/4.18±0.33; T1, 4.75±0.26/4.75±0.25; T2, 5.08±0.19/5.08±0.19; T3, 5.26±0.15/5.26±0.15; T4, 5.36±0.11/5.37±0.12; T5, 5.45±0.09/5.48±0.11; and T6, 4.9±0.24/4.89±0.22. *P* values compared to T0; T1: *p* = 0.038, T2: *p* = 0.024, T3:*p* < 0.01, T4: *p* < 0.01, T5: *p* < 0.01,T6:*p* = 0.032.

In the comparisons of the correlation between the right and left eye ONSD values ​​measured while the patient was in the neutral position and the values ​​after Trendelenburg positioning, there was a moderate-level correlation between T0 and T2 (pearson’s r:0.69/0.64 (right eye/left eye)), between T4 (Trendelenburg 75th minute) (pearson’s r:0.55/0.57) and T5 (Trendelenburg 90th minute) (pearson’s r:0.61/0.58); a high-level correlation between T0 and T3 (Trendelenburg 60th minute) (pearson’s r:0.72/0.71) and between T3 and T5 (pearson’s r:0.76/0.69) (; and a very high-level correlation between T4 and T5(pearson’s r:0.90/0.86).

Intraoperative variables, including hemodynamic and respiratory variables are shown in Table 2. When the rSO_2_ values were compared between the right and left sides, no statistically significant difference was detected in any of the measurements (*p* > 0.05). There were also no statistically significant differences in the comparison of the awake values ​​of the right and left rSO_2_ and the other values (*p* > 0.05). No statistical difference was detected in all measurements in terms of MAP, HR and ETCO2 values compared to the T0 value (*p* > 0.05).

**Table 2:** Intraoperative variables

	T0	T1	T2	T3	T4	T5	T6
R-ONSD (mm)	4.18 ± 0.32	4.75 ± 0.26*	5.08 ± 0.19*	5.26 ± 0.15*	5.36 ± 0.11*	5.45 ± 0.09*	4.9 ± 0.24*
L-ONSD (mm)	4.17 ± 0.33	4.75 ± 0.25*	5.08 ± 0.19*	5.26 ± 0.15*	5.36 ± 0.12*	5.48 ± 0.11*	4.89 ± 0.22*
MAP (mmHg)	106,8 ± 12.2	92,04 ± 8.6	93,52 ± 9.2	87,52 ± 7.4	88,44 ± 8.3	83,8 ± 9.8	82,52 ± 10.6
ETCO2 (mmHg)	34 ± 1.23	35 ± 1.05	35 ± 2.03	34 ± 3.1	37 ± 1.54	36 ± 1.87	35 ± 1.66
HR (beats/min)	75.36 ± 9.5	69.32 ± 8.6	70.24 ± 7.3	67.88 ± 7.7	68.64 ± 9.6	68.40 ± 8.5	73.92 ± 9.5
rSO2-R (%)	64,88 ± 4.5	73,4 ± 2.6	64,2 ± 3.6	66,4 ± 2.8	67,56 ± 3.5	68,46 ± 4.2	69,64 ± 2.8
rSO2-L (%)	66,40 ± 4.2	76,6 ± 2.3	67,36 ± 3.7	66,52 ± 2.2	66,52 ± 3.8	69,58 ± 3.5	70,88 ± 1.6

Values are mean ± SD. R-ONSD: Right eye-optic nerve sheath diameter; Left-ONSD: Left eye-optic nerve sheath diameter; MAP: mean arterial pressure; HR: heart rate; ETCO2: end-tidal CO2 partial pressure; rSO2-R: regional cerebral oxygen saturation-right; rSO2-L: regional cerebral oxygen saturation-left; T0. baseline (before anesthesia induction), T1. the fifth minute following endotracheal intubation in the neutral position, T2. 30 min after introducing pneumoperitoneum and Trendelenburg position, T3. 60 min after introducing pneumoperitoneum and Trendelenburg position, T4. 75 min after introducing pneumoperitoneum and Trendelenburg position, T5. 90 min after introducing pneumoperitoneum and Trendelenburg position, T6. five minutes after the patient was placed in the neutral position; * *P* < 0.05 compared with T0

There were no complications during the follow-up of the patients in the recovery room or ward.

## Discussion

In this study, we evaluated the effect of the Trendelenburg position applied during laparoscopic hysterectomy on the increase in ICP by measuring ONSD. In the results of our study, we found that the ONSD value increased as the Trendelenburg position duration increased. This suggests that as the duration of Trendelenburg position and pneumoperitoneum increases, the sustainability of the mechanisms that balance the increase in ICP becomes insufficient.

Ultrasonographic ONSD measurement, which has seen a surge in popularity in recent years, is a faster, less harmful, portable, and more practical method compared to computed tomography and magnetic resonance imaging. Anatomically, the optic nerve is an extension of the central nervous system. It is surrounded by the arachnoid, dura mater, and CSF. The optic nerve sheath is adjacent to the dura mater, and its contents are closely associated with the subarachnoid space. The intra-orbital segment of the optic nerve is surrounded by the subarachnoid space and dura, extending from the intracranial space to the globe [10]. The average diameter of the optic nerve is approximately 4 mm when including the sheath and 3 mm without the sheath. The subarachnoid space contains approximately 0.1 ml of CSF. Cases where ONSD is larger than 5 mm are consistent with increased ICP. Kimberly et al. showed that ICP was > 20 mmHg when ONSD was above 5 mm [6]. Similarly, Soldatos et al. determined that the optimal cut-off value of ONSD that accurately predicted intracranial hypertension (ICP > 20 mmHg) was 5.7–6.0 mm [5]. In another study, it was reported that during laparoscopic prostatectomy, the ONSD increased to approximately 5.0 mm after the patients were placed in the isolated upright Trendelenburg position, and this reflected a high ICP defined as 20 mmHg. However, it was observed that the upright Trendelenburg position did not accurately reflect increased ICP, despite the presence of a possible increase in ICP that could reach critical levels in some patients [11]. In the current study, we found the ONSD value to be 5.08 mm (*p* = 0.024) for both eyes in the first measurement taken 30 min after the patients were placed in the Trendelenburg position. The ONSD value was above 5.08 mm for both eyes in all measurements performed in the Trendelenburg position during surgery.

In our study, it was observed that ONSD measured at the fifth minute after intubation increased by 13.4% compared to the first value measured when the patients were awake. This increase in ONSD can be attributed to the early post-intubation phase as well as the use of mechanical ventilation and PEEP.

According to the Monro-Kellie doctrine, in the confined and rigid structure of the brain, every increase in pressure or volume in one area is counterbalanced by a reduction in another region, in order to maintain equilibrium. Thus, an increase in intracranial blood volume caused by the head-down position is attempted to be balanced with the outflow of CSF [12]. To assess the adequacy of these mechanisms, Verdonck et al. used ONSD in their study to detect increased ICP in patients undergoing laparoscopic radical prostatectomy. The authors reported no significant change in ONSD during the 190-minute Trendelenburg position period, nor did they detect any significant change in cerebral perfusion pressure with the increase in central venous pressure [9]. In contrast, in our study, we found that the ONSD value measured at the 30th minute after pneumoperitoneum and Trendelenburg position was found to be 21% higher than the initial value. As the Trendelenburg position duration increased, the increase in ONSD continued. When the Trendelenburg position duration reached 90 min, the increase in ONSD was 32.8% when compared to the initial value and 7.3% compared to the measurement performed 30 min after Trendelenburg positioning.

Schramm et al. conducted a study to investigate the time-dependent effect of the extreme Trendelenburg position applied during robotic prostate surgery on cerebral autoregulation. The authors calculated cerebral autoregulation using the cerebral blood flow rate and MAP values ​​and reported that cerebral autoregulation was impaired after the 160th minute but improved when the posture was corrected. It was also reported that keeping MAP pressure at high values during the operation could trigger or aggravate the formation of brain edema [13]. In our study, ONSD started to increase after the 30th minute of the Trendelenburg positioning, increased further as the duration of this position increased, and started to decrease upon returning to the neutral position. However, throughout the Trendelenburg position, the MAP value was maintained within the range of 80–100 mmHg.

NIRS is a noninvasive monitoring method used to measure cerebral oxygen saturation. It has been reported that the rSO_2_ value related to cerebral oxygen saturation does not change when the Trendelenburg position and pneumoperitoneum are applied to patients during surgery, and that there is a low possibility of a decrease in oxygen saturation in the frontal cortex for a duration of up to 265 min [14–16]. Consistently, in our study, we did not observe any deterioration in the rSO_2_ values ​​of any of the patients during surgery. Although ONSD increased during the Trendelenburg position period, the rSO_2_ values ​​remained stable. Matsuoka et al. reported that MAP was the main factor affecting rSO_2_ during the Trendelenburg position and pneumoperitoneum [14]. The preservation of rSO_2_ in our patients can be attributed to the MAP exceeding 80 mmHg. The Trendelenburg position may cause venous stasis and affect the arterial-venous content of the blood flowing to the forehead skin. Davie et al. reported that extracranial contamination potentially affected rSO_2_ [17]. In addition, it is important to remember that NIRS only reflects the status of the frontal region. We did not observe any postoperative neurological complications and/or cognitive impairment in any of our patients. Similarly, Kim et al. reported that while they observed a 12.5% ​​increase in ONSD in patients who underwent laparoscopic radical prostatectomy, there was no deterioration in rSO_2_ or neurological complications [18].

Our study has certain limitations. First, we did not perform central venous monitoring on our patients because we did not deem it necessary for a surgical procedure. Second, we did not perform cerebral perfusion pressure measurements. Lastly, although NIRS monitoring was undertaken to monitor cerebral oxygenation, extracranial contamination was not taken into account.

In conclusion, when the ONSD measurement is used to assess the increase in ICP during the Trendelenburg position and pneumoperitoneum applied for laparoscopic hysterectomy, the ONSD value increases as the duration of these applications increases. To prevent postoperative neurological complications, it is recommended to limit the duration of the Trendelenburg position and pneumoperitoneum.

## Data Availability

Dear editorial, The datasets used and analyzed during the current study are available from the corresponding author upon reasonable request.Best regards.

## Abbreviations

ANOVA: One-way analysis of variance
ASA: American Society of Anesthesiologists
BMI: Body mass index
CSF: Cerebrospinal fluid
ETCO2: End tidal carbon dioxide
ICP: Intracranial pressure
MAP: Mean arterial pressure
NIRS: Near infrared spectrometry
ONSD: Optic nerve sheath diameter
PEEP: Positive end-expiratory pressure
rSO2: Regional tissue oxygen saturation
